# Structural and functional studies of a noncanonical Dicer from *Entamoeba histolytica*

**DOI:** 10.1038/srep44832

**Published:** 2017-03-20

**Authors:** Xiang Yu, Xuhang Li, Lina Zheng, Jinbiao Ma, Jianhua Gan

**Affiliations:** 1 State Key Laboratory of Genetic Engineering, Collaborative Innovation Center of Genetics and Development, Department of Physiology and Biophysics, School of Life Sciences, Fudan University, Shanghai, China; 2 State Key Laboratory of Genetic Engineering, Collaborative Innovation Center of Genetics and Development, Department of Biochemistry, Institute of Plant Biology, School of Life Sciences, Fudan University, Shanghai, China

## Abstract

RNaseIII proteins are dsRNA-specific endonucleases involved in many important biological processes, such as small RNA processing and maturation in eukaryotes. Various small RNAs have been identified in a protozoan parasite *Entamoeba histolytica. Eh*RNaseIII is the only RNaseIII endonuclease domain (RIIID)-containing protein in *E. histolytica*. Here, we present three crystal structures that reveal several unique structural features of *Eh*RNaseIII, especially the interactions between the two helixes (α1 and α7) flanking the RIIID core domain. Structure and sequence analysis indicate that *Eh*RNaseIII is a noncanonical Dicer and it lacks a dsRBD in the C-terminal region (CTR). *In vitro* studies suggest that *Eh*RNaseIII prefers to bind and cleave longer dsRNAs, generating products around 25 nucleotides in length. Truncation of the CTR or attaching the dsRBD of *Aquifex aeolicus* RNaseIII can enhance the binding and cleavage activities of *Eh*RNaseIII. In combination with *in vitro* crosslinking assay, our results suggested that *Eh*RNaseIII functions in a cooperative mode. We speculate that some partner proteins may exist in *E. histolytica* and regulates the activity of *Eh*RNaseIII through interaction with its CTR. Our studies support that *Eh*RNaseIII plays an important role in producing small RNAs in *E. histolytica*.

Ribonuclease III (RNaseIII) proteins are metal-ion-dependent, double-stranded (ds) RNA-specific endonucleases^1^ that are highly conserved in bacteria and eukaryotes. In higher eukaryotes, RNaseIII proteins, such as Dicer and Drosha, play important roles in the RNA interference (RNAi) pathway^2^. Dicer converts long dsRNAs into small interfering RNA (siRNA) duplexes that are 21–25 nucleotides (nt) long with a phosphate group at the 5′-end and a 2-nt overhang at the 3′-end^3^,^4^. These features are critical for the loading of siRNA duplexes onto the RNA-induced silencing complex (RISC)^5^,^6^. Argonaute (Ago) is the effector protein of RISC and it is activated after the degradation of the siRNA passenger strand^7^. The guide strands of siRNA duplexes then direct the RISC to the target RNAs via Watson–Crick base pairing^8^,^9^. Similar to the passenger strands, the target RNAs are cleaved by RISC, leading to their silencing. In lower eukaryotes that do not have the RNAi system, such as *Saccharomyces cerevisiae*, RNaseIIIs play multiple roles in the processing and maturation of precursor rRNAs, small nucleolar RNAs, and small nuclear RNAs^10^. In bacteria, RNaseIIIs are mainly involved in rRNA maturation and post-transcriptional gene regulation^11^.

Based on their sizes and domain architectures (Fig. 1A), RNaseIIIs can be divided into four classes^1^,^12^. Class I RNaseIIIs mainly exist in bacteria, such as *Escherichia coli* RNaseIII (*Ec*RNaseIII)^13^ and *Aquifex aeolicus* RNaseIII (*Aa*RNaseIII)^14^,^15^,^16^,^17^. Class I RNaseIIIs are around 230 aa in size and they possess one N-terminal RIIID domain and one C-terminal dsRBD. Compared with class I RNaseIIIs, class II RNaseIIIs are longer (~500 aa). Class II RNaseIIIs are represented by *Saccharomyces cerevisiae* Rnt1 (*Sc*Rnt1)^18^,^19^ and *Kluyveromyces polysporus* Dicer 1 (*Kp*Dcr1); both have an N-terminal extension domain (NTD) that forms an intermolecular dimer. As revealed by the crystal structure of *Sc*Rnt1-product complex^18^, the NTD domain forms several hydrogen bonds (H-bond) with the AGUC tetraloop of the RNA substrate. Deletion of the NTD lowers the RNA processing accuracy of *Sc*Rntp1. Class III RNaseIIIs are represented by *Homo sapiens (Hs*) Drosha^20^,^21^. *Hs*Drosha is around 1,400 aa long and possesses one P-rich and one RS-rich domain at the N-terminus followed by one platform and one PAZ-like domain in the middle, and two RIIID domains and one dsRBD domain at the C-terminus. Class IV RNaseIIIs are represented by *Hs*Dicer^22^,^23^,^24^, which is close to 1,900 aa in length. Similar to *Hs*Drosha, *Hs*Dicer also contains two RIIID domains followed by one dsRBD domain at the C-terminus. The N-terminus of *Hs*Dicer is composed of one helicase domain and one DUF283 domain followed by a platform and a PAZ domain, and these domains are involved in the binding and terminus recognition of substrate RNAs^9^. Extensive structural and functional studies have been carried out for these representative RNaseIIIs, including *Aa*RNaseIII, *Sc*Rnt1, and *Hs*Drosha, which elucidated the catalytic mechanism and structural basis for substrate recognition; however, the structures and functions of many non-representative RNaseIIIs from other species remain elusive.

*Entamoeba histolytica* is a protozoan parasite that infects millions of people and causes nearly 100,000 amoebiasis deaths worldwide per year, according to a World Health Organization report of 1997^25^. *Entamoeba histolytica* has two life-cycle stages: the cyst form and the trophozoite form. The cyst form is its dormant stage that helps the parasite survive adverse conditions. In the trophozoite form, the parasite can infect people and cause disease^26^. Various small RNAs, with lengths of 16 nt, 22 nt, and 27 nt, have been discovered in *E. histolytica*^27^. There are three genes in the *E. histolytica* genome (*EHI_186850*, *EHI_125650*, and *EHI_177170*) that encode Ago proteins. Among them, Ago2-2, encoded by *EHI_125650*, is highly expressed and associates with the 27-nt RNAs^27^,^28^,^29^. The *E. histolytica* genome also contains a homolog of the RNA-dependent RNA polymerases (RdRP), which are essential for small RNA biogenesis in some eukaryotes, such as *S. pombe, C. elegans*, and plants. Interestingly, there is only one RNaseIII protein (*Eh*RNaseIII) encoded by the *E. histolytica* genome^30^, which is composed of 256 amino acids (aa). *in vivo* RNaseIII activity has been detected in *E. histolytica* trophozoites^31^ and, recently, it was confirmed that *Eh*RNaseIII can process dsRNA in the RNAi-negative background of *Saccharomyces cerevisiae*^32^, and it can partially reconstitute the RNAi pathway in conjunction with *Saccharomyces castellii* Ago1^33^. These observations suggest that *Eh*RNaseIII may play a role in the RNAi pathway in *E. histolytica*.

To further investigate the potential role of *Eh*RNaseIII and to uncover the structural basis underlying its functions, we performed crystallographic studies and *in vitro* catalytic assays of *Eh*RNaseIII (Supplementary Fig. S1). Herein, we present three high-resolution crystal structures of *Eh*RNaseIII, including selenomethionine (SeMet)-labeled *Eh*RNaseIII (aa 1–194, SeMet-*Eh*RNaseIII194), *Eh*RNaseIII229 (aa 1–229), and an *Eh*RNaseIII229-Mn^2+^ complex. These structures, in combination with sequence analysis, suggest that *Eh*RNaseIII is a noncanonical Dicer that possesses some very unique structural features. Our *in vitro* assays show that the C-terminal region (CTR) of *Eh*RNaseIII has an inhibitory effect on its binding and cleavage of dsRNA, and that this effect can be attenuated by removal of the CTR or by attaching a classical dsRNA binding domain (dsRBD) after the CTR. *Eh*RNaseIII preferentially binds and cleaves longer dsRNAs, generating products of around 25 nt. Based on these observations, we propose that *Eh*RNaseIII binds and cleaves dsRNAs in a cooperative way, and we speculate that some unknown partner proteins, most likely dsRBD-containing proteins, may exist in *E. histolytica* that can enhance the activity of *Eh*RNaseIII.

## Results

### *Eh*RNaseIII is a non-canonical Dicer

**Figure 1:**
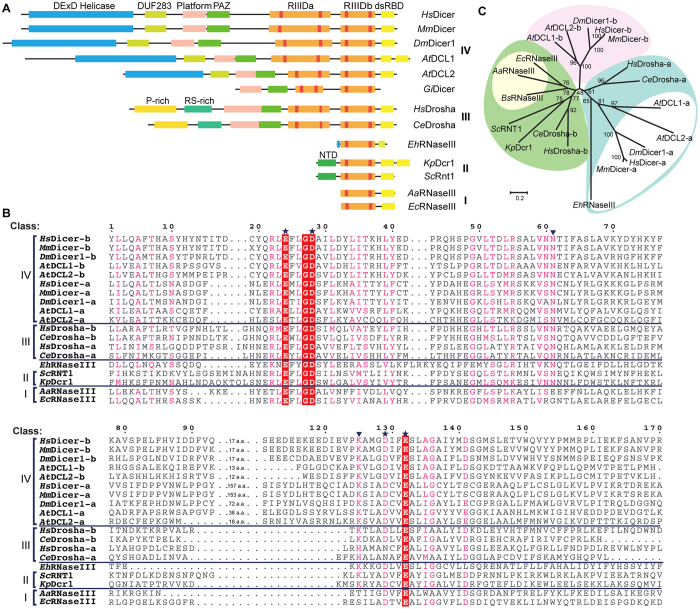
EhRNaseIII is a non-canonical Dicer. (**A**) Domain architectures of representative RNaseIIIs; tandem RIIID domains of Drosha and Dicer are designated as RIIIDa and RIIIDb, respectively. *Hs*, *Homo sapiens*; *Mm*, *Mus musculus*; *Dm, Drosophila melanogaster*; *At, Arabidopsis thaliana*; *Gi, Giardia intestinalis*; *Ce, Caenorhabditis elegans*; *Sc, Saccharomyces cerevisiae; Kp, Kluyveromyces polysporus*; *Eh, Entamoeba histolytica*; *Aa*, *Aquifex aeolicus*; *Ec*, *Escherichia coli*. (**B**) Sequence alignment of RIIID domains from each class of RNase III enzyme. *Hs*Dicer1, GI: 152012889; *Hs*Drosha, GI: 20139357; *Mm*Dicer, GI: 257051057; *Dm*Dicer1, GI: 7300916; *At*DCL1, GI: 34922211; *At*DCL2, GI: 332640405; *Ce*Drosha, GI: 20141625*; Sc*RNT1, GI: 618855177; *Kp*Dcr1, GI: 342351115; *Eh*RNaseIII, GI: 56467134; *Aa*RNaseIII, GI: 160877684; *Ec*RNaseIII, GI: 485668531. Conserved catalytic residues are indicated by asterisks, newly identified catalytic residues are labeled with an inverted triangle. (**C**) Maximum-likelihood tree of RIIID domains from RNaseIII families. RIIIDa and RIIIDb domains of class IV RNaseIIIs are colored white and pink, respectively. RIIIDa and RIIIDb domains of class III RNaseIIIs are colored cyan and green, respectively. Class II RNaseIIIs are also colored green. Class I RNaseIIIs are colored orange. *Bs, Bacillus subtilis*.

The common structural features of all RNaseIIIs are the RIIIDs (Fig. 1B). RIIID is characterized by a signature motif, which is _38_ERLEFLGD_46_ in *Ec*RNaseIII; E41 and D46 are two conserved catalytic residues. Two more negatively charged residues (corresponding to D114 and E117 in *Ec*RNaseIII) are also highly conserved and critical for the catalytic activity of RNaseIIIs, although in some class III RNaseIIIs there is a D → N variation in the first RIIID domain (RIIIDa) (Fig. 1B). Although *Eh*RNaseIII has very low similarity to these representative RNaseIIIs, sequence alignment was able to identify the signature motif (_48_EKNEFYGD_55_) and the two conserved catalytic residues (D116 and E119). Sequence alignment also identified two more conserved residues (N91 and K112) in *Eh*RNaseIII. These two residues are not conserved in bacterial RNaseIIIs; whereas they are highly conserved throughout eukaryotic RNaseIIIs (with a K → H variation in the RIIIDa domains of *Ce*Drosha and *Hs*Drosha). *In vitro* studies in *Hs*Dicer and *Kp*Dcr1 showed that N → A and K → A mutation will significantly reduce the cleavage activities of the proteins, suggesting that these two residues play important roles during the cleavage reaction^34^.

The conservation of the six catalytically important residues suggests that *Eh*RNaseIII is closely related to the eukaryotic RNaseIIIs. Evolutionary analysis further indicates that *Eh*RNaseIII is similar to the RIIIDa domains of Drosha and Dicer proteins (Fig. 1C). However, the size of *Eh*RNaseIII is more similar to the bacterial RNaseIIIs compared with the eukaryotic RNaseIIIs. The size of the CTR (corresponding to aa 163–256) of *Eh*RNaseIII is similar to the typical bacterial RNaseIII dsRBDs, but no sequence similarity was identified between them. A typical dsRBD, such as the dsRBDs of *Aa*RNaseIII, adopts an αβββα fold (Supplementary Fig. S2). The second α-helix sits in-between the first α-helix and the three β-strands, and it plays two functionally important roles: it stabilizes the dsRBD structure, and enhances dsRBD and dsRNA binding through the formation of H-bonds. According to the secondary structure prediction program GOR4, there are two consecutive α-helixes followed by two short β-strands in the *Eh*RNaseIII CTR region. Phylogenetic analysis and the lack of a dsRBD domain suggest that *Eh*RNaseIII might represent a noncanonical Dicer.

### Overall structure of *Eh*RNaseIII

Three *Eh*RNaseIII structures were solved in this study, including SeMet-*Eh*RNaseIII194, *Eh*RNaseIII229, and *Eh*RNaseIII229-Mn^2+^ complex. The structures all belong to P2_1_2_1_2_1_ space groups with one *Eh*RNaseIII intermolecular dimer per asymmetric unit. The C-terminal 24 residues (aa 196–229) were disordered in the *Eh*RNaseIII229 structure; all structures are very similar with rmsd (root mean square deviations) of 0.4–0.7 Å between all *Eh*RNaseIII dimers. Because of its high resolution, the *Eh*RNaseIII229 structure was used for structural analysis and comparison hereafter.

**Figure 2:**
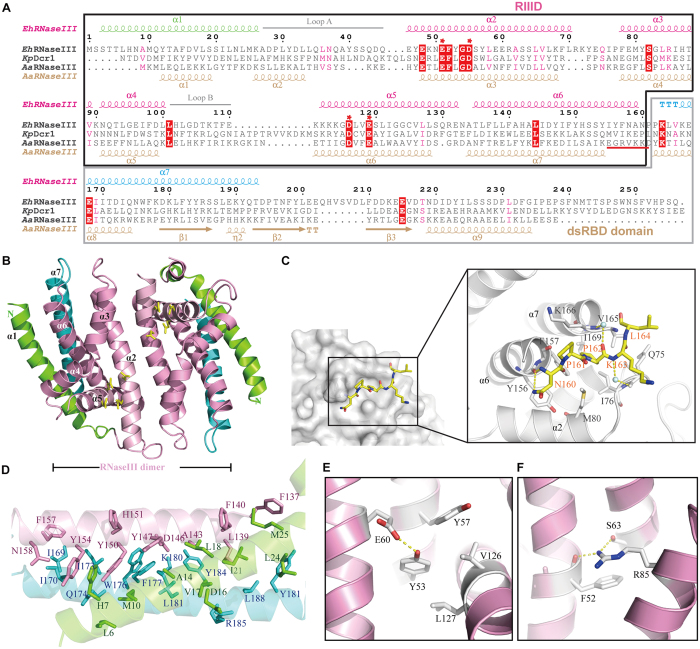
Sequence alignment and crystal structure of EhRNaseIII. (**A**) Structure-based sequence alignment. The secondary structures of *Eh*RNaseIII and *Aa*RNaseIII are shown on the top and bottom, respectively. The 100% conserved residues are highlighted with red background. Residues involved in catalysis are marked by red asterisks. The RIIID domains are marked by a black box and the dsRBDs are indicated with a gray box. (**B**) Overall structure of *Eh*RNaseIII229. Helix α1, RIIID core, and helix α7 are colored green, pink, and blue, respectively. The catalytic site residues are colored yellow. (**C**) The conformation of the α6–α7 linker between RIIID and CTR. The linker is shown in stick format in atomic colors (C, yellow; N, blue; O, red), water molecules were shown as spheres in pale cyan. The hydrogen bonds are indicated with yellow dashed lines. (**D**) Interactions between α1, RIIID core, and α7. The side chains are shown in stick format in green, pink, and blue for α1, RIIID core, and α7, respectively. (**E**) and (**F**) show the interactions that may stabilize the *Eh*RNaseIII dimer from the back and catalytic sites, respectively.

Each *Eh*RNaseIII229 monomer contains seven helixes (Fig. 2A,B); the conformations of α1 (aa 1–28) and α7 (aa 165–195) are unique, compared with other RNaseIII structures, including *Aa*RNaseIII, *Kp*Dcr1, and *Sc*Rnt1. In most of the RNaseIII structures, there is a flexible linker between the RIIID and the dsRBD domains, which provides the structural basis for the major conformational changes associated with substrate binding (Supplementary Fig. S2). As confirmed by the *Ec*RNaseIII study, substitution of Q153 by the rigid P153 residue in the middle of the linker will reduce the linker flexibility and abolish the dsRNA cleavage activity^35^. α6(aa 135–159) corresponds to the last helix in other RIIID domain structures, and it is connected to α7 through a 5-residue linker (referred as the α6–α7 linker, _160_NPPKL_164_). Unlike other RNaseIIIs, the α6–α7 linker of *Eh*RNaseIII forms tight interactions with the surrounding residues (Fig. 2C). Via the N and ND2 atom, N160 forms two H-bonds (2.8 Å and 3.1 Å) with the O atom of Y156. The side chain of P162 sits in a hydrophobic pocket formed by I76, M80, F157, V165, and I169; the backbone O atom of P162 interacts with K166 via H_2_O-mediated H-bond. The H_2_O-mediated H-bond was also observed between K163 and Q75. Although P161 does not form direct interactions with other residues, it could further reduce the flexibility of the linker owing to its rigidity.

α7 was fixed in the structure, it forms several interactions with α1 (Fig. 2D), including the hydrophobic interactions formed by the side chains of M10, F177, and L181, one H-bond (3.2 Å) between the O atom of S3 and the NE2 atom of Q174, and one salt bridge (2.5 Å) between the OD2 atom of D16 and the NH2 atom of R185. Both α1 and α7 were further stabilized by their interactions with α6. The N-terminus of α6 (_135_TLFLLFAHALI_145_) mainly interacts with α1 and α7 through hydrophobic interactions. Besides hydrophobic interactions, the C-terminus of α6 (_147_YIFYHSSYIYFNA_159_) also forms several H-bonds with α1 and α7, via the OH groups of Y147 and Y150, the ND1 atom of H151, and the OD1 atom of N158. In between the N-terminus and C-terminus of α6, there is one charged residue, D146. Interestingly, the OD2 atom of D146 forms one salt bridge (2.8 Å) with the NZ atom of K180, and one H-bond (2.6 Å) with the OH group of Y184, respectively. Other helixes, such as α2, also interact with α1 and α7.

The conformations of two loops, loop A (_32_DLLQLNQAYSS_42_, the loop between helixes α1 and α2) and loop B (_103_LGDTKTFE_110_, the loop between helixes α4 and α5), are significantly different in the *Eh*RNaseIII229 and the SeMet-*Eh*RNaseIII194 structures. The conformation of loop B is also different in the *Aa*RNaseIII and *Kp*Dcr1 structures (Supplementary Figs S3A and C). In the *Aa*RNaseIII-RNA complex structure, loop B interacts with the major groove of dsRNA (Supplementary Fig. S3B). Loop B of *Eh*RNaseIII is shorter than the corresponding loops of *Aa*RNaseIII and *Kp*Dcr1 by 5 and 12 aa, respectively (Fig. 2A), and it may contribute to the weak dsRNA-binding ability of *Eh*RNaseIII described later on.

### Unique RIIID core domain

For efficient RNA cleavage, RNaseIIIs have to form a dimer either intramolecularly or intermolecularly. Although *Eh*RNaseIII229 contains seven helixes, structural comparison revealed that the RIIID core domain only contains the middle five helixes, α2–α6. As depicted in Fig. 2B, the dimer interface of *Eh*RNaseIII229 is mainly formed by α2 (aa 42–71) and α3 (aa 78–89). There are some other dimerization-enhancing interactions, such as helixes swapping or loop cross-talking in the *Kp*Dcr1 and *Aa*RNaseIII structures, respectively; however, such interaction was not observed in the *Eh*RNaseIII229 structure. α2 contains the signature motif [_48_EKNEFYGD_55_, which has three highly conserved residues (underlined)]. In all other RNaseIIIs, there is another highly conserved residue (Leu or Val) prior to the conserved Gly and Asp residues, whereas it is a Tyr residue (Y53) at the corresponding position in *Eh*RNaseIII. The OH atom of Y53 forms one very tight H-bond (2.6 Å) with the OE2 atom of E60 from the partner molecule (Fig. 2E); and together with its hydrophobic interactions with the surrounding residues, such as Y57, L67, V126, and L127, Y53 may function as a lock holding the two monomers together from the opposite site of the catalytic valley. Interestingly, there is another lock at the catalytic valley side (Fig. 2F), which is composed of F52, S63, and R85. The NH1 atom of the R85 side chain forms two H-bonds, one (3.0 Å) with the O atom of the F52 backbone, and another (3.0 Å) with the OG atom of the S63 side chain. In addition, F52 and R85 also interact with each other through the stacking of their side chains.

**Figure 3:**
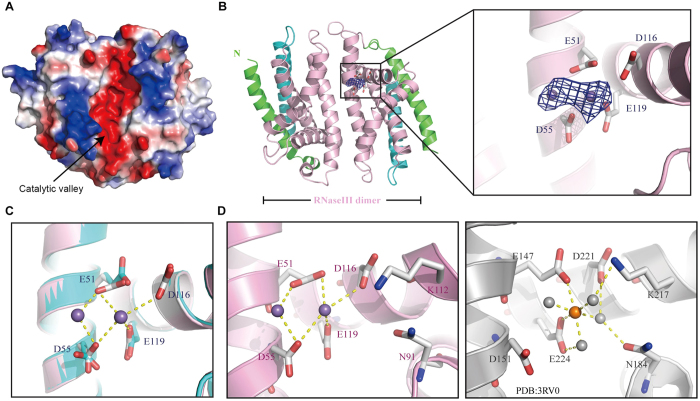
Coordination of Mn2+ ions. (**A**) The surface presentation of *Eh*RNaseIII. The highly electronegative catalytic valley is indicated by black arrows. (**B**) The overall structure of the *Eh*RNaseIII-Mn^2+^ complex. The side chains of catalytic residues are shown in stick format in atomic colors (C, gray; O, red), Mn^2+^ ions are shown as spheres outlined with the F_o_-F_c_ omit map (contoured at the 3.0 σ level). (**C**) Structural superposition showing the conformational differences in the presence and absence of Mn^2+^ ions. The *Eh*RNaseIII-Mn^2+^complex structure was colored using the color scheme used in (**B**). The backbone of *Eh*RNaseIII in the apo-*Eh*RNaseIII structure is shown as a cartoon in cyan, the side chains of the catalytic residues are shown in stick format in atomic colors (C, cyan; O, red). Coordinations between Mn^2+^ ions and the catalytic residues are indicated with yellow dashed lines. (**D**) Structural comparison between the active sites of *Eh*RNaseIII (left panel) and *Kp*Dcr1 (PDB_ID: 3RV0, right panel). Mn^2+^ ion and the coordinating water molecules in the *Kp*Dcr1 structure are shown as spheres in orange and gray, respectively.

The rmsd between the core RIIID domain of *Eh*RNaseIII229 and that of the *Aa*RNaseIII (aa 18–145) is 2.8 Å, and is 2.3 Å and 2.1 Å, when compared with the core RIIID domains of *Kp*Dcr1 (aa 112–260) and *Sc*Rnt1 (aa 197–363), respectively. As shown in Fig. 3A, the catalytic valley of *Eh*RNaseIII is highly negatively charged; actually, it is more negatively charged when compared with *Aa*RNaseIII, *Kp*Dcr1, and *Sc*Rnt1 structures, owing to the presence of E59 (which resides right next to the two-fold axis of the dimer). E59 is not conserved in the class I and class II RNaseIIIs, but it is highly conserved in the RIIIDa domains of class III RNaseIIIs and the RIIIDb domains of class IV RNaseIIIs (Fig. 1B), though the functional importance of this residue remains elusive.

### Conserved two metal ion binding site

The active site of *Eh*RNaseIII (Fig. 3B) contains four negatively charged residues (E51, D55, D116, and E119), which form two metal-binding sites: the prominent metal-binding site (M1) and the second metal-binding site (M2). Divalent metal ions (preferentially Mg^2+^ and Mn^2+^) are required for the RNA cleavage reaction catalyzed by RNaseIIIs. The complex structures have been determined for RNaseIIIs from different classes, such as *Aa*RNIII-Mg^2+^ (PDB code: 1RC5) in class I^36^, and *Kp*Dcr1-Mg^2+^ (PDB code: 3RV0, Fig. 3D) in class II. Both *Aa*RNIII-Mg^2+^ and *Kp*Dcr1-Mg^2+^ complex structures were obtained through co-crystallization method, which uses 1.5 mM MgCl_2_ in the protein sample and 20 mM MgCl_2_ in the crystallization buffer, respectively. Very surprisingly, although the crystallization buffer contains 20 mM MgCl_2_, no Mg^2+^ ion was bound at the M1 or M2 sites in the *Eh*RNaseIII229 structure, suggesting that the Mg^2+^ ion-binding affinity of *Eh*RNaseIII is weak. The *Eh*RNaseIII229-Mn^2+^ complex structure (Fig. 3B) was obtained by soaking the *Eh*RNaseIII229 crystals overnight in crystallization buffer supplemented with 10 mM MnCl_2_. The occupancy of Mn^2+^ ions at the catalytic site A was very low, so it was not modeled in the structure. In contrast to catalytic site A, two well-defined Mn^2+^ ions were bound at the M1 and M2 positions of catalytic site B. As depicted in Fig. 3C, the M1 site Mn^2+^ ion coordinates with the side chains of E51 and D55; whereas the Mn^2+^ ion at the M2 site coordinates with E51, D116, and E119. Structural comparison revealed that the conformations of D116 and E119 are conserved; whereas, E51 and D55 can undergo obvious conformational changes upon binding of Mn^2+^ ions.

In eukaryotic RNaseIIIs, there are two more important conserved catalytic residues, one Asn and one Lys (Fig. 3D). There are four consecutive Lys residues (K111-K114) in *Eh*RNaseIII, and structural superimposition revealed that K112 is the important one for catalysis. The backbone of K112 is well defined, but the side chain is very flexible, indicated by the extremely weak electron density. In the *Kp*Dcr1 structure, the NZ atom of K217 forms one H-bond (2.5 Å) with the nucleophilic water, which attacks the phosphorus atom at the cleavage site^34^. In the *Sc*Rnt1 structure, the NZ atom of K313 forms one H-bond (2.9 Å) with the OP1 atom of the product 5′-phosphate group^18^. These differences suggest that the flexibility of the K112 side chain is functionally relevant and that it provides the structural basis for the necessary conformational changes associated with the metal ion and RNA binding. In the *Kp*Dcr1 structure, N184 interacts with the Mg^2+^ ion at the M1 site through one water molecule (the distance between the bridge water and the OD1 atom of N184 is 2.9 Å). Both M1 and M2 sites were occupied by an Mg^2+^ ion in the *Sc*Rnt1 structure; interestingly, the M2 site Mg^2+^ ion also interacted with N278 through a water molecule, and the distance between the bridge water and OD1 atom of N278 is 2.6 Å. These interactions indicated that the conserved Asn residue was mainly involved in the stabilization of the metal ions. N91 residues are very stable in all our *Eh*RNaseIII structures, supported by the well-defined electron densities. Structural comparison further reveals that the overall conformations of N91 in *Eh*RNaseIII structures are similar to that of N184 in *Kp*Dcr1 (Fig. 3D) and N278 in the *Sc*Rnt1-product structure.

### *Eh*RNaseIII CTR affects dsRNA binding

**Figure 4:**
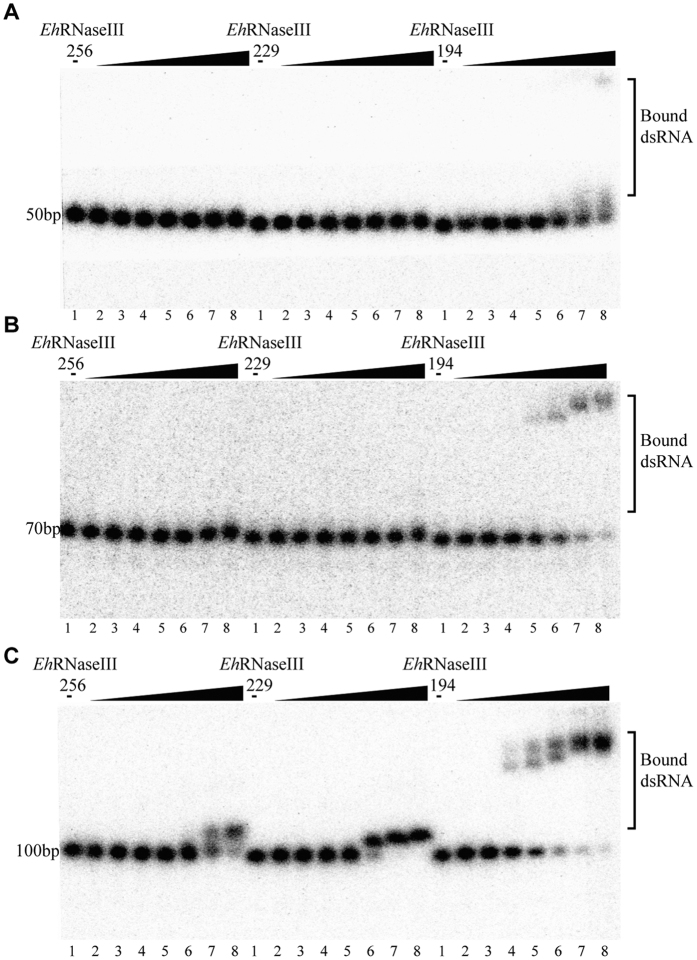
dsRNA binding by EhRNaseIII256, EhRNaseIII229, and EhRNaseIII194. (**A**) RNA50, (**B**) RNA70, and (**C**) RNA100 are dsRNAs with lengths of 50, 70, and 100 bp, respectively. The RNAs were incubated without (Lane 1) or with *Eh*RNaseIIIs (Lane 2–8). The concentrations of *Eh*RNaseIIIs are 10^−6^ M, 10^−5^ M, 5 × 10^−5^ M, 10^−4^ M, 3 × 10^−4^ M, 6 × 10^−4^ M, and 10^−3^ M in Lanes 2–8, respectively.

In the *Eh*RNaseIII229 structure, the α7 (aa 165–195) folds back and forms tight interactions with N-terminal α1 and other helixes of the RIIID core; the remaining 61 residues (aa 196–256) are too short to form a typical dsRBD, which is consistent with the secondary structure prediction results. For other RNaseIIIs, such as *Ec*RNaseIII and *Kp*Dcr1, their dsRBDs play an important role in the dsRNA substrate-binding and cleavage reaction. To better understand the functional role of the *Eh*RNaseIII CTR, EMSA assays (Fig. 4) were carried out using different dsRNA substrates and various *Eh*RNaseIII proteins, including *Eh*RNaseIII194, *Eh*RNaseIII229, and *Eh*RNaseIII256. In total, four sets of dsRNA substrates, RNA25, RNA50, RNA70, and RNA100 were used in the EMSA. Among them, RNA25 was not bound by all three *Eh*RNaseIII proteins (not shown). *Eh*RNaseIII256 did not bind RNA50 or RNA70 (Fig. 4A,B, left panel), whereas, it did bind RNA100 (Fig. 4C, left panel). The apparent K_d_ for the RNA100 binding by *Eh*RNaseIII256 was ~6 × 10^−4^ M, which is much lower than that of *Kp*Dcr1^34^. *Eh*RNaseIII229 did not bind RNA50 or RNA70 (Fig. 4A,B, middle panel); however, similar to *Eh*RNaseIII256, *Eh*RNaseIII229 could bind RNA100 (Fig. 4C, middle panel). Interestingly, the RNA100-binding affinity of *Eh*RNaseIII229 was at least 2-fold higher than that of *Eh*RNaseIII256, as revealed by the almost complete shifting of RNA100 in lane 6 (the concentration of *Eh*RNaseIII229 was 3 × 10^−4^ M). The lower K_d_ of *Eh*RNaseIII229 protein suggests that the C-terminal aa 230–256 have an inhibitory effect on dsRNA substrate binding.

*Eh*RNaseIII194 can bind all dsRNA substrates, including RNA50, RNA70, and RNA100. Although its binding affinity to RNA50 is still low (Fig. 4A, lane 8 of the right panel), *Eh*RNaseIII194 can bind RNA70 substrate at the concentration of 1.0 × 10^−4^ M (Fig. 4B, lane 5 of the right panel), and this binding is tighter than the binding between RNA100 and *Eh*RNaseIII256 (also at 1.0 × 10^−4^ M concentration). RNA100 can be shifted by *Eh*RNaseIII194 at the concentration of 5.0 × 10^−5^ M (Fig. 4C, lane 4 of the right panel); this binding affinity is about 6- and 2-fold higher than that of *Eh*RNaseIII256 and *Eh*RNaseIII229, respectively, estimated from the molar ratios of bound RNAs versus free RNAs. These observations indicate that aa 195–229 also play a role in inhibiting dsRNA substrate binding.

The dsRNA binding affinity of *Eh*RNaseIII194 follows the order: RNA100>RNA70>RNA50>RNA25 (Supplementary Fig. S4), indicating that the binding affinity is correlated with the substrate size. A similar conclusion can also be drawn for *Eh*RNaseIII229 and *Eh*RNaseIII256, based on the EMSA results depicted in Fig. 4. *Eh*RNaseIII256 and *Eh*RNaseIII229 form one major complex with RNA100, which moves just slightly slower than the free RNAs. However, such complex was not observed in the case of *Eh*RNaseIII194; instead, *Eh*RNaseIII194 forms two complexes with the RNAs, and both of them move much more slowly than the complexes formed in the presence of *Eh*RNaseIII229 and *Eh*RNaseIII256. These observations suggest that the slow moving complex may contain multiple *Eh*RNaseIII194 dimers.

### *in vitro Eh*RNaseIII cleavage activity

dsRNA cleavage activities of RNaseIIIs are dependent on the divalent metal ions, preferentially Mg^2+^. *Eh*RNaseIII has a conserved RIIID, including the conserved residues that coordinate with the metal ions. No Mg^2+^ ion was observed at the catalytic site of *Eh*RNaseIII structures; however, previous studies have revealed that the metal ion (especially the one at the M2 position) binding affinities of RNaseIIIs can be enhanced by the presence of RNA substrates. Therefore, it is possible that *Eh*RNaseIII is still active in the presence of Mg^2+^. To explore this possibility, we carried out *in vitro* cleavage assays with RNA substrates in the presence of Mg^2+^; however, very surprisingly, no detectable dsRNA cleavage activity was observed for any *Eh*RNaseIII proteins (Supplementary Fig. S5A), indicating that Mg^2+^ alone was not enough to assemble the *Eh*RNaseIII-dsRNA complex in catalytic form.

**Figure 5:**
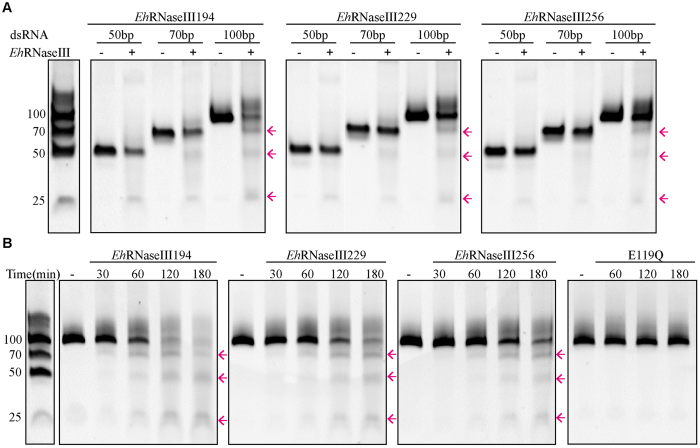
*in vitro* dsRNA cleavage catalyzed by EhRNaseIIIs. (**A**) The cleavage of RNA50, RNA70, and RNA100 by *Eh*RNaseIII256, *Eh*RNaseIII229, or *Eh*RNaseIII194. (**B**) The RNA100 cleavage assay with time course. dsRNAs were incubated without (−) or with *Eh*RNaseIIIs at a concentration of 10^−5^ M, the concentration of Mn^2+^ is 10 mM for all reactions. The reaction mixtures were incubated for 100 min in (**A**), and the detailed reaction time were labelled on the figure in (**B**). The product RNAs were indicated by pink arrows. The gels were cropped from the original images available at the Supplementary Fig. S6.

Some RNaseIIIs are also active in the presence of Mn^2+^; and, as for other cation-dependent nucleases, our structure revealed that the binding of the negatively charged catalytic residues with Mn^2+^ was stronger than that with Mg^2+^. Therefore, we also carried out the cleavage assay in the presence of Mn^2+^. Almost no RNA25 was cleaved by the three native proteins, including *Eh*RNaseIII256, *Eh*RNase229, and *Eh*RNase194 (not shown); whereas, the RNA50, RNA75, and RNA100 could be cleaved by all three proteins under the same reaction conditions (37 °C, 100 min). As exampled by *Eh*RNase194 (Fig. 5A, left panel), the major cleavage product of RNA50 is about 25 nt in size; there are two major products formed in the case of RNA70, which are about 25 nt and 50 nt, respectively. Besides these two products, another product with a length close to the 70-nt marker was generated from RNA100. As indicated by the product intensity, the RNA cleavage activity of *Eh*RNase194 follows the order: RNA100>RNA70>RNA50; similar results are also observed for *Eh*RNase229 (Fig. 5A, middle panel) and *Eh*RNaseIII256 (Fig. 5A, right panel), suggesting that *Eh*RNaseIII preferentially cleaves the longer RNAs. Interestingly, besides the product bands, some slow moving bands were observed on the gel; these bands may be caused by the *Eh*RNaseIII proteins, which are not completely denatured under the condition.

The RNA100 cleavage activity of the proteins follows the order: *Eh*RNase194>*Eh*RNase229>*Eh*RNase256; though it is not as obvious as RNA100, the proteins follow the same order in cleaving RNA70 and RNA50 (Fig. 5A). These observations suggest that the CTR of *Eh*RNaseIII may have certain inhibitory effect on substrate cleavage, and this conclusion can be further supported by the *in vitro* cleavage assay of RNA100 with time course. As depicted in Fig. 5B, there are significant amount of products generated at the reaction time of 60 min for *Eh*RNase194; whereas only small amount of products formed in the presence of *Eh*RNase229 and only trace amount of products is observed in the case of *Eh*RNase256. As the reaction time was increased, more substrates are cleaved by the proteins; the longest products are converted into the shorter ones. Though it needs to be further determined, the pattern and the convergence of these products suggests that the sizes of the two longer fragments might be double and triple that of the smallest one, which is about 25-nt in size. As a negative control, the *in vitro* cleavage assay with the catalytic deficient mutant E119Q was also carried out; as depicted in Fig. 5B, no any product generated, confirming that the above *Eh*RNaseIII cleavage activities are not caused by contamination.

### dsRNA binding and cleavage activities are enhanced in the chimeric protein EA256

Many small RNAs exist in *E. histolytica*, and *Eh*RNaseIII is the only RNaseIII protein identified in *E. histolytica*. As revealed by our *in vitro* studies, *Eh*RNaseIII is active in the presence of Mn^2+^ ions, but very high protein concentrations (up to the μM level) are required for efficient substrate cleavage. The C-terminal dsRBDs play an important role in the dsRNA processing by *Kp*Dcr1; removing the two dsRBDs dramatically reduces the cleavage activity and results in the formation of heterogeneous products^34^. Some other RNaseIIIs also have dsRBD-containing protein partners (Supplementary Fig. S8), which play critical roles in miRNAs biogenesis, such as DGCR8 for Drosha in *Homo sapiens*^37^, HYL1 for DCL1 in *Arabidopsis thaliana*^38^, and Loqs-PD and R2D2 for Dicer2 in *Drosophila*^39^. Like the dsRBDs of *Kp*Dcr1, these partner proteins also have dsRNA-binding ability.

**Figure 6:**
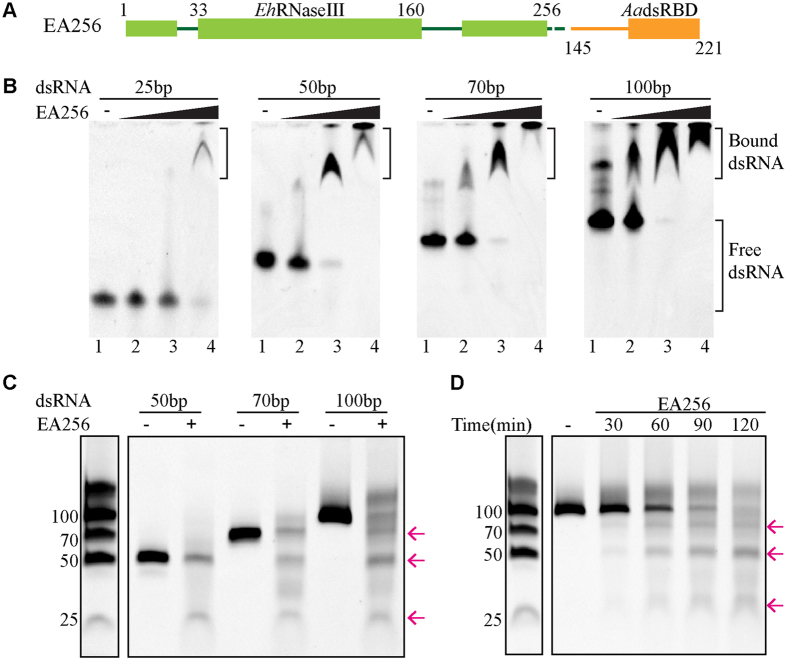
dsRNA binding and cleavage by chimeric protein EA256. (**A**) The schematic depicts the domain architecture of EA256. EA256 is composed of full-length *Eh*RNaseIII and the dsRBD domain of *Aa*RNaseIII, which are colored green and orange, respectively. (**B**) Binding of dsRNAs by EA256. dsRNAs were incubated without (−) or with EA256 (Lanes 2–4). The EA256 concentrations are 10^−6^ M, 10^−5^ M, and 10^−4^ M, in Lanes 2, 3, and 4, respectively. (**C**) The cleavage of RNA50, RNA70, and RNA100 by EA256. (**D**) The RNA100 cleavage assay with time course. dsRNAs were incubated without (−) or with 10^−6^ M EA256, the Mn^2+^ concentration is 10 mM for all reactions. The reaction mixtures were incubated for 100 min in (**C**), and the detailed reaction time were labelled on the figure in (**D**). The product RNAs are indicated by pink arrows. The gels were cropped from the original images available at the Supplementary Fig. S7.

*Eh*RNaseIII does not contain a dsRBD domain; and to test whether a dsRBD can enhance the RNA-binding and cleavage activity of *Eh*RNaseIII, we constructed a chimeric *Eh*RNaseIII protein, EA256 (Fig. 6A), which is composed of *Eh*RNaseIII and the dsRBD domain of *Aa*RNaseIII. The *Aa*RNaseIII dsRBD was selected because its structure and its interaction with dsRNAs have been well characterized^14^,^15^,^16^,^17^ (Supplementary Fig. S2). As shown in Fig. 6B, EA256 can bind RNA50, RNA70, and RNA100 completely at the concentration of 1.0 × 10^−4^ M (Lane 4); at this concentration, EA256 can also bind more than 70% of RNA25, which does not interact with *Eh*RNaseIII256. These results suggest that EA256 has significantly improved dsRNA-binding affinity and compared with *Eh*RNaseIII256, the binding affinity was estimated to be increased by 10- to 100-fold.

Similar to *Eh*RNaseIII256, EA256 is not active in the presence of Mg^2+^ ions (Supplementary Fig. S5B). In the presence of Mn^2+^, the dsRNA cleavage activity of EA256 is much higher than the *Eh*RNaseIII256; at the concentration of 1.0 × 10^−6^ M, EA256 can efficiently digest all the RNA substrates, including RNA50, RNA70, and RNA100 (Fig. 6C). As estimated from the gel, about 20% RNA50 was cleaved at the reaction time of 100 min, created a product, which is about 25 nt in size; under the same condition, more than 50% RNA70 was cleaved, formed two products with lengths about 25 and 50 nt, respectively. Degraded by EA256, one product with longer length was also observed in the case of RNA100; the pattern of these product bands are very similar to those of *Eh*RNaseIII256, *Eh*RNaseIII229, and *Eh*RNaseIII194. Also similar to these *Eh*RNaseIII proteins, EA256 can convert the longer product into the short ones when the reaction time increased (Fig. 6D). These observations suggested that EA256 shares the similar substrate binding and cleavage mechanism as the native *Eh*RNaseIII proteins.

### *Eh*RNaseIII cleaves dsRNA in a cooperative mode

RNaseIIIs from higher species have developed various mechanisms to precisely control their product sizes, which is critical for their functions. The product lengths of Dicer are determined by the RNA structures and the cooperative interactions of the PAZ, dsRBD, and helicase domains^40^,^41^,^42^; *Sc*Rnt1 uses two molecular rulers embedded at the NTD and dsRBD domains to ensure accurate cleavage of the substrate^18^. Due to lack of molecular ruler, the products created by class I RNaseIIIs vary in sizes. As revealed by the *Aa*RNaseIII structure, the product could be as short as 11 nt. Similar to the class I RNaseIIIs, *Eh*RNaseIII has no known molecular ruler. However, the obvious product pattern (Figs 5, 6C and D) suggests that *Eh*RNaseIII can control its product length via certain method.

Similar to *Eh*RNaseIII, no molecular ruler exists in *Kp*Dcr1; however, previous study revealed that *Kp*Dcr1 can achieve the precise substrate cleavage through the cooperative interactions between the protein molecules^34^. It was proposed that *Kp*Dcr1 dimers bind the conjugated dsRNA along its length, and two variable loops (VL-1 and VL-2) within the RIIIDs play an important role in the packing of neighboring dimers. VL-1 corresponds to the loop that extends along the RNA minor groove in the *Aa*RNaseIII-product structure (PDB code: 2NUG); replacement of VL-1 with the analogous regions from the *Gi*Dicer RIIIDb domain would reduce its RNA cleavage activity and generate heterogeneous products. *Kp*Dcr1VL-2 corresponds to the loop that constitutes the RNA-binding motif 4 in *Aa*RNaseIII; substitution of VL-2 with the analogous regions from the *Gi*Dicer RNIIIb domain would completely abolish the dsRNA cleavage activity of *Kp*Dcr1.

**Figure 7:**
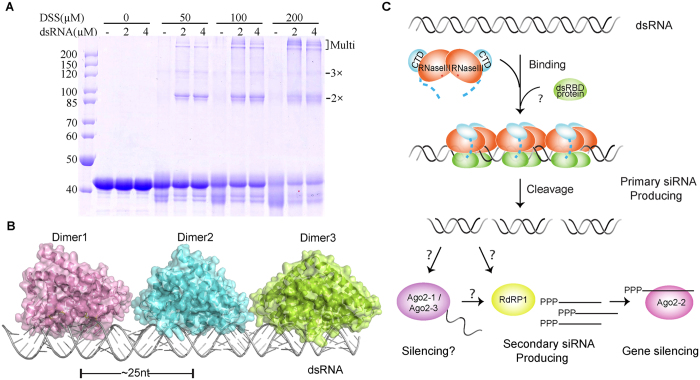
The cooperative dsRNA binding mode. (**A**) The DSS crosslinking of EA256 (20 μM) and RNA100. The RNA100 concentrations are 2 μM or 4 μM if present, the DSS concentrations were indicated on the figure. The bands corresponding to two, three, and multiple EA256 molecules were indicated by pink arrows. (**B**) Three *Eh*RNaseIII dimers modeled with long dsRNA showing the cooperative binding between *Eh*RNaseIII and dsRNA. (**C**) Proposed model for activation and dsRNA cleavage by *Eh*RNaseIII. Multiple *Eh*RNaseIIIs can bind a long dsRNA in a cooperative mode. Some unknown cofactor, which functions as a dsRNA-binding protein, enhances the dsRNA binding and cleavage. The length of the RNA products is ~25 nt. Some siRNAs detected in *E. histolytica* possess a tri-phosphate group at their 5′-ends, which may result from the secondary siRNA pathway. RdRP encoded by the *E. histolytica* genome may participate in the amplification of the tri-phosphate modified siRNAs, which will be loaded onto Ago2-2 and lead to silencing of the target gene. Other siRNAs created by *Eh*RNaseIII may be recognized by another two Ago proteins, Ago2-1 and Ago2-3, and cause gene silencing.

To verify whether *Eh*RNaseIII cleaves the substrates via the cooperative model similar to *Kp*Dcr1, the protein-RNA crosslinking assay was carried out using RNA100 and EA256, due to its higher activity. As depicted in Fig. 7A, RNAs alone has no impact on the shifting of EA256 on the gel. *Eh*RNaseIII functions as dimer, however, DSS (Disuccinimidyl suberate, the crosslinking reagent) alone does not lead to the formation of the dimer bands, may due to the lack of proper lysine residues on the dimerization interface. Interestingly, some faster moving bands appeared at the bottom of the gel, which may be caused by the DSS modification on the *Eh*RNaseIII monomer. When both RNAs and DSS are present, several bands corresponding to two, three, or multiple *Eh*RNaseIII molecules appeared. The Loop A (aa 32–42) and Loop B (aa 103–110, Supplementary Fig. S3) of *Eh*RNaseIII correspond to the VL-1 and VL-2 loops of *Kp*Dcr1, respectively. Interestingly, the loop B of *Eh*RNaseIII is shorter than VL-2 by 12 nt; it is also 5 nt shorter than the corresponding loops in *Aa*RNaseIII. As revealed by our *Eh*RNaseIII structures, the loop A and loop B are flexible and they can undergo large conformational changes. Tough it needs to further verified whether the loop A and loop B are involved in the substrate binding, our crosslinking assay clearly indicated that *Eh*RNaseIII functions through the cooperative mode.

In the *Aa*RNaseIII-product structure, *Aa*RNaseIII dimers were adjacently packed along the pseudocontinuous dsRNA formed by 11-nt RNAs, and the distance between the two active sites of the adjacent RNaseIII dimers was 22 nt. The size of dsRNA products generated by *Kp*Dcr1 was 23 nt, whereas the products were about 25 nt in size in the presence of *Eh*RNaseIII, suggesting that the RNA-binding mode of *Eh*RNaseIII may not be exactly same as that of *Aa*RNaseIII and *Kp*Dcr1. To better illustrate the cooperative dsRNA binding mode, we built a *Eh*RNaseIII-dsRNA binding model, depicted in Fig. 7B.

## Discussion

*Eh*RNaseIII is the only RIIID-containing protein identified in *E. histolytica*. As revealed by our structural studies, *Eh*RNaseIII lacks a typical dsRBD in its CTR and is a noncanonical Dicer protein. *Eh*RNaseIII possesses some very unique structural features, including the cross-talking between helixes α1 and α7, and a unique dimerization enhancing mechanism. *Eh*RNaseIII has a conserved RIIID core; and as revealed by *in vitro* catalytic assays, *Eh*RNaseIII is active in the presence of Mn^2+^ and can produce RNA product with a length ~25 nt. These results indicate that *Eh*RNaseIII may play a role during the siRNA biogenesis process in *E. histolytica*.

The size of the small RNAs identified in *E. histolytica* varies and includes RNAs of 16, 22, and 27 nt. Unlike the RNA products generated in the *in vitro* assay, which possess a monophosphate group at the 5′-end, many of the small RNAs discovered in *E. histolytica* have a triphosphate group at their 5′-end, which is similar to siRNAs found in *C. elegans.* In *C. elegans*, the 5′-triphosphate capped siRNAs are amplified by the RdRP-dependent secondary siRNA production pathway^43^,^44^, suggesting that the *E. histolytica* siRNAs are not the immediate products of *Eh*RNaseIII cleavage. Besides *Eh*RNaseIII and Ago proteins, other homologous proteins involved in the secondary RNAi pathway, such as RdRP, also exist in *E. histolytica*. We speculate that the RdRP protein may be responsible for the 5′-triphosphate formation (Fig. 7C). In the RNAi pathway in higher eukaryotes, many RNaseIIIs have dsRBD-containing protein partners, such as DGCR8 partnering *Hs*Drosha and TRBP partnering *Hs*Dicer, which play critical roles in small RNA biogenesis. No *Eh*RNaseIII cleavage activity was observed in the presence of Mg^2+^ in our *in vitro* assay; whereas, previous studies showed that Mg^2+^ can support the cleavage activity of *Eh*RNaseIII in cell lysate^43^,^44^. The functions of many of the proteins expressed in *E. histolytica* remain to be characterized, and we speculate that one or more of dsRBD-containing proteins may function as an *Eh*RNaseIII partner. These partner proteins may regulate the dsRNA-binding and cleavage activity of *Eh*RNaseIII *in vivo* via their interaction with the CTR of *Eh*RNaseIII. Also, with the help of the partner proteins, *Eh*RNaseIII should be functional in the presence of Mg^2+^, which is the physiological cofactor of many known RNaseIII proteins.

## Materials and Methods

### Plasmid construction

The plasmid used for overproduction of the recombinant His-Sumo-*Eh*RNaseIII was constructed as follows. The full-length gene of the wild-type *Eh*RNaseIII was PCR amplified from *E. histolytica* cDNA using two primers, *Eh*RNaseIII-BamHI-1F and *Eh*RNaseIII-SalI-256R. The product was double-digested with BamHI and SalI, and cloned into the Sumo-tag-containing pET28 vector (Novagen), referred as pET28-Sumo hereafter. Then, the plasmid was transfected into *Escherichia coli* strain BL21(DE3) and its sequence was confirmed through DNA sequencing. The CTR truncated proteins *Eh*RNaseIII194 and *Eh*RNaseIII229 were constructed using the same procedure but with primer *Eh*RNaseIII-SalI-256R replaced with *Eh*RNaseIII-SalI-194R and *Eh*RNaseIII-SalI-229R, respectively. The E119Q mutant was constructed using the site-direct mutagenesis method with two primers: *Eh*RNaseIII-E119Q-F and *Eh*RNaseIII-E119Q-R; the plasmid of the full-length wild type His-Sumo-*Eh*RNaseIII was used as template. The DNA construct of chimeric protein *Eh*RNaseIII-*Aa*dsRBD (EA256, which contains the full-length *Eh*RNaseIII followed by the dsRBD of *Aa*RNaseIII) was generated by overlapping PCR. The *Eh*RNaseIII and *Aa*RNaseIII plasmids served as templates for two PCR reactions: (1) with primers *Eh*RNaseIII-BamHI-1F and *Eh*RNaseIII-256R-Aa, and (2) with primers *Aa*RBD-145F and *Aa*RBD-221-SalI-R, respectively. The amplicons of PCR reactions 1) and 2) were combined and used as template for a third PCR reaction with primers *Eh*RNaseIII-BamHI-1F and *Aa*RBD-221-SalI-R. The resulting amplicon was cloned into the pET28-Sumo vector and transfected into *E. coli* BL21(DE3) competent cells for DNA sequencing and protein expression. A schematic diagram of the *Eh*RNaseIII constructs is depicted in Supplementary Fig. S1 and the detailed sequences of the primers are listed in Supplementary Table S1.

### Protein expression and purification

All *Eh*RNaseIII proteins were expressed and purified using identical procedures, as described below. Each recombinant strain was cultured at 37 °C in 1 L LB medium supplemented with 50 μg/mL kanamycin, and protein expression was induced at OD_600_ ≈ 0.6 by the addition of isopropyl β-D-1-thiogalacto-pyranoside (IPTG, final concentration 0.2 mM). The induced culture was then grown at 18 °C overnight. The cells were harvested by centrifugation and resuspended in the lysis buffer (20 mM Tris-HCl pH 8.0, 500 mM NaCl, 25 mM imidazole pH 8.0). The cells were lysed under high pressure using a JNBIO homogenizer (Guangzhou Juneng Biology & Technology Co., Ltd.). The homogenate was clarified by centrifugation, and the supernatant was loaded onto a HisTrap^TM^ HP column (GE Healthcare) and eluted with elution buffer (20 mM Tris-HCl pH 8.0, 500 mM NaCl, 500 mM imidazole pH 8.0) using a linear gradient. The fractions containing the recombinant His-Sumo-*Eh*RNaseIII protein were pooled and digested with Ulp1 protease while being dialyzed against buffer S (20 mM Tris-HCl pH 8.0, 500 mM NaCl). The protein was loaded onto a HisTrap^TM^ HP column again to remove the cleaved His-Sumo tag. The flow-through containing the target *Eh*RNaseIII protein was diluted to lower the NaCl concentration to 100 mM and was then loaded onto a HiTrap Q column equilibrated with buffer A (20 mM Tris-HCl pH 8.0, 100 mM NaCl). The protein samples were eluted with buffer B (20 mM Tris-HCl pH 8.0, 1 M NaCl) using a linear gradient. The eluted sample was concentrated and loaded onto a HiLoad 16/60 Superdex^TM^ 75 column (GE Healthcare) equilibrated with gel filtration buffer (10 mM Tris-HCl pH 8.0, 100 mM NaCl). Protein was concentrated using an Amicon-Ultra centrifugal device from Millipore and its purity was analyzed by SDS-PAGE.

For the overproduction of selenomethionine (Se-Met) substituted *Eh*RNaseIII194 protein (SeMet-*Eh*RNaseIII194), the *E. coli* BL21(DE3) strain containing the recombinant pET28-Sumo-*Eh*RNaseIII194 plasmid was grown in 100 mL LB medium supplied with 50 μg/mL kanamycin at 37 °C overnight. Next day, the cells were pelleted and resuspended in 2 L M9 medium and grown at 37 °C. When the culture reached the early log phase (OD_600_ ≈ 0.6), the temperature was lowered to 18 °C. One hour later, 0.2 mM IPTG and 60 mg/L Se-Met (J&K) were added to induce the protein expression. The induced culture was then grown at 18 °C overnight. The cells were pelleted by centrifugation and the SeMet-*Eh*RNaseIII194 protein was purified using the same procedures as those used for the native proteins. DTT (1 mM) was present in all purification buffers to avoid the oxidation of Se.

### Crystallization and X-ray diffraction data collection

Crystals of SeMet-*Eh*RNaseIII194 were grown using the hanging-drop vapor diffusion method at 16 °C. The droplets contained equal volumes of protein sample and reservoir solution [1.26 M (NH_4_)_2_SO_4_, 0.1 M Hepes pH 7.8]. Crystals of *Eh*RNaseIII229 were obtained using the sitting-drop vapor diffusion method in a 3-drop intelliplate at 16 °C. The drop contained 0.3 μL protein solution and 0.3 μL crystallization buffer [1.6 M (NH_4_)_2_SO_4_, 0.02 M MgCl_2_, 0.05 M Tris-HCl pH 7.5]. To obtain the *Eh*RNaseIII229-Mn^2+^ complex crystals, the freshly grown *Eh*RNaseIII229 crystals were transferred sequentially into crystallization buffer supplemented with 5 mM and 10 mM MnCl_2_ and soaked for 30 min in each solution. Then, the crystals were transferred into crystallization buffer containing 10 mM MnCl_2_ and 20% glycerol and soaked overnight. The soaked crystals were frozen by plugging directly into liquid nitrogen. Crystals of SeMet-*Eh*RNaseIII194 and *Eh*RNaseIII229 were cryoprotected by dipping quickly into their mother liquid supplemented with 20% glycerol and flash frozen in liquid nitrogen.

All the X-ray diffraction data were collected on beamline BL17U and BL19U at the Shanghai Synchrotron Radiation Facility at the cryogenic temperature maintained by the cryogenic system. One single crystal was used in each case. The Se-Met single*-*wavelength anomalous diffraction data of SeMet-EhRNaseIII194 was collected at a wavelength of 0.97915 Å. Other data were all collected at a wavelength of 1.0000 Å. All data processing was carried out with the HKL2000 program^45^ and the data collection and processing statistics are summarized in Table 1.

### Structure determination and refinement

The SeMet-*Eh*RNaseIII194 structure was solved using the Se-Met single*-*wavelength anomalous diffraction method^46^ with the AutoSol program^47^ embedded in the PHENIX suit^48^; the Figure of Merit value was 0.49. The program identified six out of the eight incorporated Se atoms and generated an initial model that covered more than 75% of protein residues in the asymmetric unit. The side chains of the residues were manually built based on the electron density map using the graphic program, Coot^49^. The partial model was then refined against the diffraction data using the Refmac5 program embedded in CCP4i^50^. The more complete model of SeMet*-Eh*RNaseIII194 was built based on the improved density map resulting from the refinement. The *Eh*RNaseIII229 and *Eh*RNaseIII229-Mn^2+^ complex structures were solved by the molecular replacement method using the Phaser^51^ program of CCP4i; the SeMet*-Eh*RNaseIII194 structure was used as the search model. The resulting model was refined using Refmac5 and the phenix.refine program^52^ of PHENIX. During refinement, 5% of randomly selected data was set aside for free R-factor cross validation calculations. The 2F_o_-F_c_ and F_o_-F_c_ electron density maps were regularly calculated and used as guides for the building of the missing residues. Water molecules were added either automatically or manually using Coot. Sulfate and metal ions were modeled in the refinement until the last few cycles. The R_work_ and R_free_ were 0.202 and 0.246, 0.207 and 0.240, and 0.197 and 0.240 for the SeMet-*Eh*RNaseIII194, *Eh*RNaseIII229, and *Eh*RNaseIII194-Mn^2+^ structures, respectively. All the residues were located in the favored or allowed regions of the Ramachandran plot. The detailed structure refinement statistics are summarized in Table 1.

### RNA preparation

All the RNAs used in this work (Supplementary Table S1) were produced by *in vitro* transcription catalyzed by T7 RNA polymerase^53^. The pUC19 plasmids containing the target sequences were ordered from Shanghai GENERAY Biotech, amplified and purified using a Miniprep Kit (Qiagen) according to the manufacturer’s instructions. Prior to *in vitro* transcription, the templates were linearized using SmaI, extracted with phenol-chloroform, and precipitated with ethanol. The transcription reactions were carried out at 37 °C for 5 h. α-^32^P-UTP [with a 100:1 molar ratio of UTP:α-^32^P-UTP (3000 Ci/mmol)] was included during the reaction to generate ^32^P body-labeled ssRNAs in some cases. Reactions were quenched by the addition of 1 μL 0.5 M EDTA. The template DNAs were digested with DNase I, and then the samples were resolved by denaturing PAGE (using a 15% gel for 25 and 50-nt ssRNAs, and a 10% gel for 70 and 100-nt ssRNAs). UV254-shadowing (over Xerox paper) was used to visualize the RNAs. The target RNAs were eluted from gel slices with elution buffer (0.02% SDS, 1 mM EDTA, 0.3 M NaAc) overnight at 37 °C and precipitated using ethanol. ssRNAs were dissolved in RNase-free ddH_2_O and the concentrations were determined using an ultraviolet spectrometer.

dsRNAs, RNA25, RNA50, RNA70, and RNA100, which are 25, 50, 70, and 100 bp long, respectively, were generated by annealing of complementary ssRNAs. The complementary RNAs were mixed at a molar ratio of 1:1 in annealing buffer (30 mM Tris-HCl pH 7.5, 100 mM NaCl, 1 mM EDTA). The mixture was heated at 95 °C for 2 min, and slowly cooled to room temperature. Annealed dsRNAs were fractionated by native PAGE and detected by autoradiography or ultraviolet shadowing. dsRNAs were eluted form gel slices with elution buffer (0.02% SDS, 1 mM EDTA, 0.3 M NaAc), ethanol precipitated and stored in storage buffer (10 mM Tris-HCl pH 7.5, 10 mM NaCl, 0.1 mM EDTA).

### dsRNA binding assays

Binding of *Eh*RNaseIIIs, including *Eh*RNaseIII256 (the full-length *Eh*RNaseIII), *Eh*RNaseIII229, *Eh*RNaseIII194, and chimeric protein EA256, to the dsRNA substrates was monitored using electrophoretic mobility shift assays (EMSA). Five microliters of *Eh*RNaseIII, 3 μL dsRNA, and 2 μL 5 × binding buffer (150 mM Tris-HCl pH 7.5, 150 mM NaCl, 25 mM MgCl_2_, 5 mM DTT, 0.5 mM EDTA, 25% glycerol) were mixed in a thin-wall Eppendorf tube. The final concentrations of *Eh*RNaseIIIs and dsRNAs are indicated on the figures. The reaction mixtures were incubated at room temperature for 10 min followed by incubation on ice for an additional 20 min. Samples were loaded onto a pre-cooled 6% native polyacrylamide gel. Gels were run at 160 V for 3–4 h at 4 °C in 0.5× TBE buffer supplemented with 5 mM MgCl_2_. RNAs were visualized by phosphorimaging using a Typhoon 9000 (GE Healthcare) (for Fig. 4) or by staining with Gelred (Biotium) (for Figs 5 and 6).

### *Eh*RNaseIII activity assays

Ten-microliter samples consisting of 2 μL 5× reaction buffer (150 mM Tris-HCl pH 7.5, 150 mM NaCl, 50 mM MnCl_2_, 5 mM DTT, 0.5 mM EDTA), 5 μL protein and 3 μL RNA (3 μM for RNA25, and 1 μM for RNA50, RNA70, and RNA100) were mixed and incubated at 37 °C; the protein concentrations and the incubation times were given at the figure legends. Reactions were quenched by the addition of 10 μL loading buffer (90% formamide, 18 mM EDTA, 0.025% SDS, 0.02% bromophenol blue). Samples were heated at 95 °C for 5 min, centrifuged, and loaded onto 8 M urea 10% PAGE. Gels were run at 10 W for 50 min in 0.5 × TBE and stained with Gelred for 15 min. RNAs were detected using a Gel-Imaging system (Bio-Rad).

### Crosslinking assays

Four-microliter samples consisting of 1 μL 5× reaction buffer (150 mM Hepes-NaOH pH 7.6, 150 mM NaCl, 25 mM MgCl_2_, 5 mM DTT, 0.5 mM EDTA), 2 μL EA256 protein (50 μM) and 1 μL RNA (10 μM or 20 μM for RNA100) were mixed and incubated at room temperature for 30 min. One-microliter diluted DSS (disuccinimidyl suberate, sigma) were added to the reaction system with the final concentration of 50 μM, 100 μM, and 200 μM, respectively. The final concentrations of RNA100 are 2 μM or 4 μM if present. The samples were incubated at room temperature for an additional 20 min and quenched by the addition of 1.25 μL SDS-loading buffer. Samples were heated at 95 °C for 5 min, centrifuged, and loaded onto 10% SDS-PAGE. Gels were run at 200 V for 60 min in 1× SDS running buffer and stained with coomassie brilliant blue.

## Additional Information

**How to cite this article:** Yu, X. *et al*. Structural and functional studies of a noncanonical Dicer from *Entamoeba histolytica. Sci. Rep.* **7**, 44832; doi: 10.1038/srep44832 (2017).

**Publisher's note:** Springer Nature remains neutral with regard to jurisdictional claims in published maps and institutional affiliations.

## Supplementary Material

Supplementary Information

## Figures and Tables

**Table 1:** Data collection and structural refinement statistics.

	SeMet-RNaseIII194	*Eh*RNaseIII229	*Eh*RNaseIII229-Mn^2+^
Data collection			
Wavelength (Å)	0.97915	1.00000	1.00000
Space group	P2_1_2_1_2_1_	P2_1_2_1_2_1_	P2_1_2_1_2_1_
Unit cell			
a,b,c (Å)	43.3, 91.0, 102.7	46.1, 89.9, 100.3	46.3, 89.7, 100.2
α,β,γ (°)	90.0, 90.0, 90.0	90.0, 90.0, 90.0	90.0, 90.0, 90.0
Resolution rang (Å)^α^	30.0–2.10 (2.18–2.10)	30.0–1.90(1.97–1.90)	50.0–2.05 (2.12–2.05)
No. of unique observations	45330 (4553)	31630 (3234)	25785 (2666)
Completeness (%)^α^	99.1 (99.1)	94.8 (98.6)	95.3 (99.9)
*R*_sym_ (%)^α^	5.3 (19.8)	8.9 (42.5)	10.5 (45.7)
*I*/σ*I*^α^	24.2 (9.0)	26.8 (8.1)	16.4 (2.6)
Redundancy	3.8 (3.7)	6.6 (5.3)	5.5 (5.2)
Refinement			
Resolution	30.0–2.10	28.7–1.90	44.9–2.05
R_work_ (%)	20.2	20.7	19.7
R_free_ (%)	24.6	24.0	24.0
No. Of reflections	24145	31554	25617
Model quality			
Estimated coordinate error (Å)	0.21	0.27	0.25
r.m.s.d. bonds (Å)	0.007	0.008	0.003
r.m.s.d. angles (°)	0.925	0.804	0.574
Ramachandran plot (%)			
Most favored	97.1	98.9	97.6
Additional allowed	2.9	1.1	2.4
Disallowed	0	0	0

^a^Values in parentheses are for the last resolution shell.
