# Supplementary material for: Structural and functional studies of a noncanonical Dicer from Entamoeba histolytica
Source: Sci Rep. 2017 Mar 20;7:44832. doi: 10.1038/srep44832 (PMC5357909; doi:10.1038/srep44832)
Supplement: Supplementary Information [file srep44832-s1.pdf]

Supplementary data

for

Structural and functional studies of noncanonical Dicer from *Entamoeba histolytica*

Xiang Yu[1,2], Xuhang Li[2], Lina Zheng[2], Jinbiao Ma[2,*], Jianhua Gan[1,*]

[1] State Key Laboratory of Genetic Engineering, Collaborative Innovation Center of Genetics and Development, Department of Physiology and Biophysics, School of Life Sciences, Fudan University, Shanghai, China

[2] State Key Laboratory of Genetic Engineering, Collaborative Innovation Center of Genetics and Development, Department of Biochemistry, Institute of Plant Biology, School of Life Sciences, Fudan University, Shanghai, China

*Corresponding authors:

E-mail: ganjhh@fudan.edu.cn or majb@fudan.edu.cn

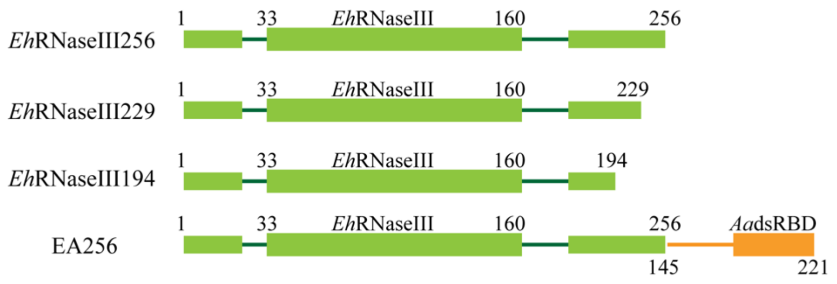

Supplementary Figure S1. The schematic representation of *Eh*RNaseIIIs constructed in this work.

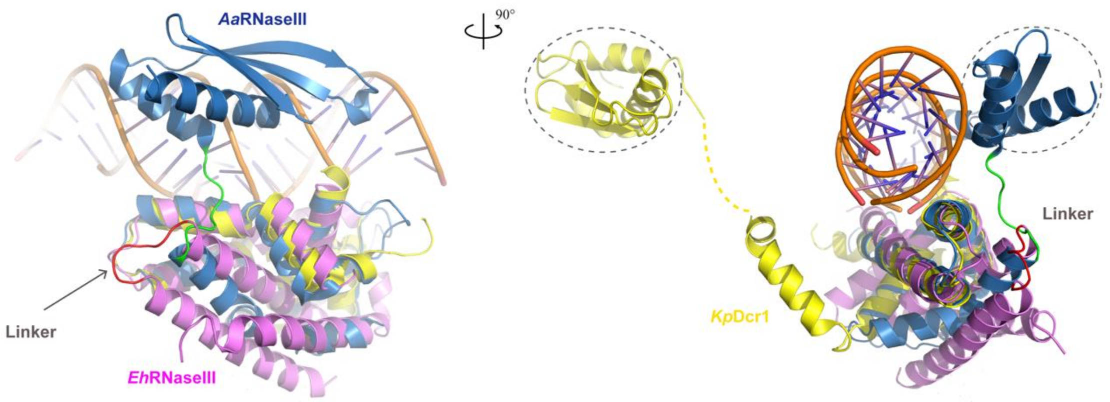

Supplementary Figure S2. Structural comparison of *Eh*RNaseIII, *Aa*RNaseIII and *Kp*Dcr1. The structures were aligned based on the RIIID. *Eh*RNaseIII, *Aa*RNaseIII and *Kp*Dcr1 are colored in magenta, blue and yellow, respectively. The $\alpha6-\alpha7$ linker of *Eh*RNaseIII is colored in red. The dsRNAs are shown as cartoon in orange and the linker connecting the RIIID and dsRBD domains is colored in green in *Aa*RNaseIII. The linker connecting the RIIID and dsRBD domains of *Kp*Dcr1 is disordered and presented by yellow dashed lines. The dsRBD domains are highlighted with gray dotted oval.

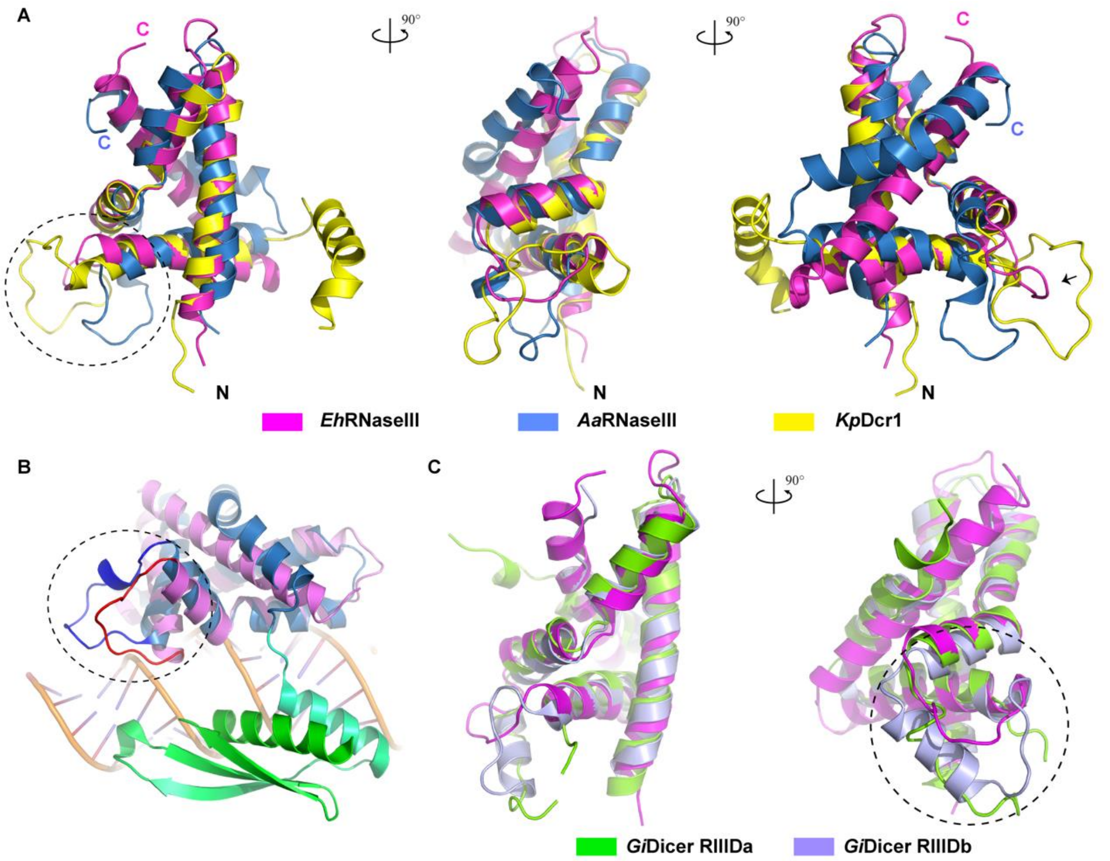

Supplementary Figure S3. The different conformations of the $\alpha4-\alpha5$ loop. (**A**) Superposition of *Eh*RNaseIII, *Aa*RNaseIII and *Kp*Dcr1, which are colored in magenta, blue and yellow, respectively. The $\alpha4-\alpha5$ loops are highlighted with black dotted oval. (**B**) Structural superposition of *Eh*RNaseIII and *Aa*RNaseIII-RNA complex. The dsRBD domain of *Aa*RNaseIII-RNA complex is colored in green. (**C**) Superposition of *Eh*RNaseIII, and *Giardia intestinalis* (*Gi*) Dicer. The RIIIDa and RIIIDb domains of *Gi*Dicer are colored in green and violet, respectively.

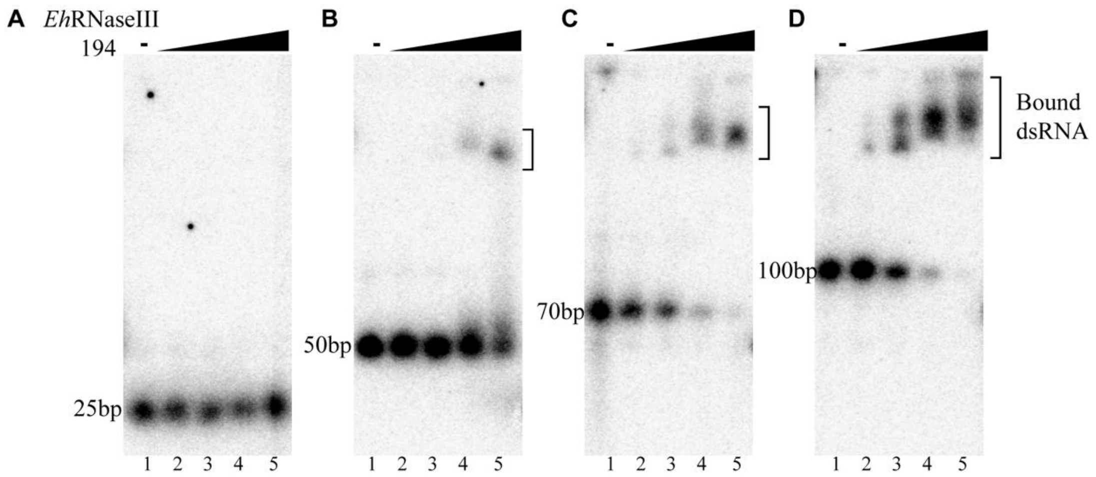

Supplementary Figure S4. The binding of RNA25 (**A**), RNA50 (**B**), RNA70 (**C**) and RNA100 (**D**), by *Eh*RNaseIII194. RNA25, RNA50, RNA70 and RNA100 are dsRNAs with length of 25bp, 50bp, 70bp and100bp, respectively. The RNAs were incubated without (Lane 1) or with *Eh*RNaseIII194 (Lane 2-5). The concentrations of *Eh*RNaseIII194 are 5×10^{-5} M, 10^{-4} M, 5×10^{-4} M and 10^{-3} M, at Lane 2-5, respectively.

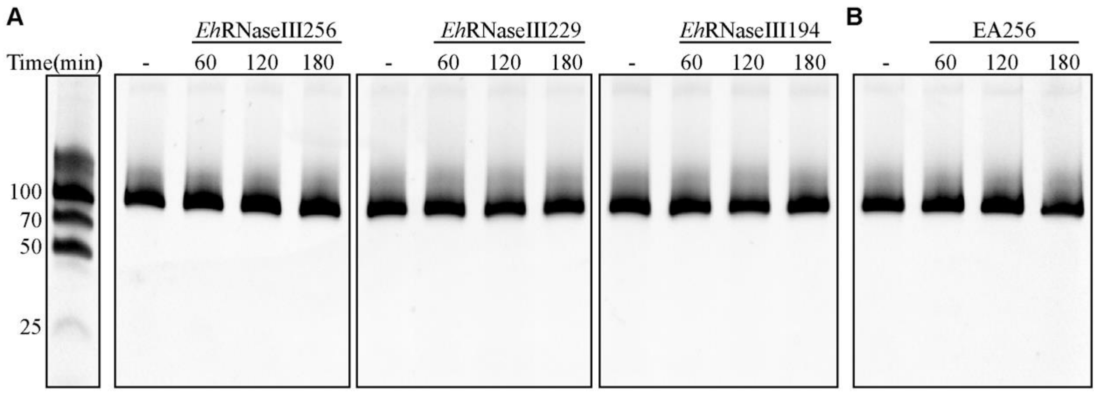

Supplementary Figure S5. *in vitro* RNA100 cleavage by *Eh*RNaseIIIs in the presence of Mg^{2+} ions (10 mM). (**A**) The assays catalyzed by 10^{-5} M *Eh*RNaseIII256, *Eh*RNaseIII229, and *Eh*RNaseIII194. (**B**) The assays catalyzed by 10^{-6} M chimeric EA256 protein. The detailed reaction times were labelled on the figure.

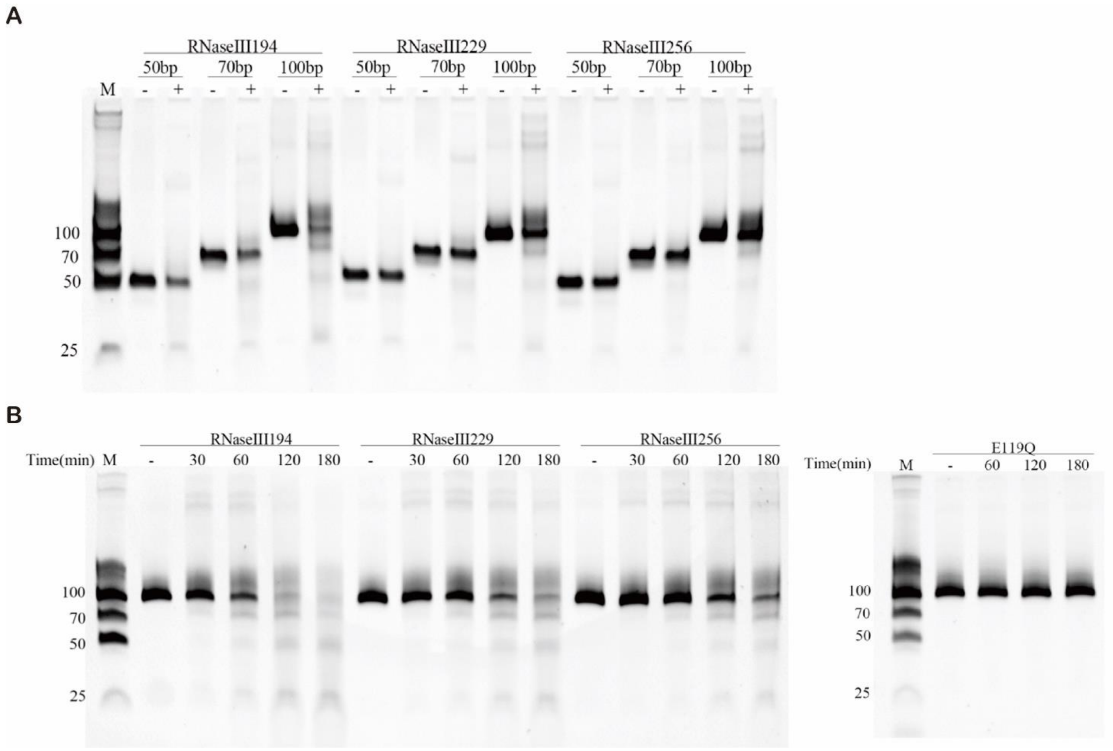

Supplementary Figure S6. (**A**) Original image of cropped gels used in Fig. 5A. (**B**) Original images of cropped gels used in Fig. 5B.

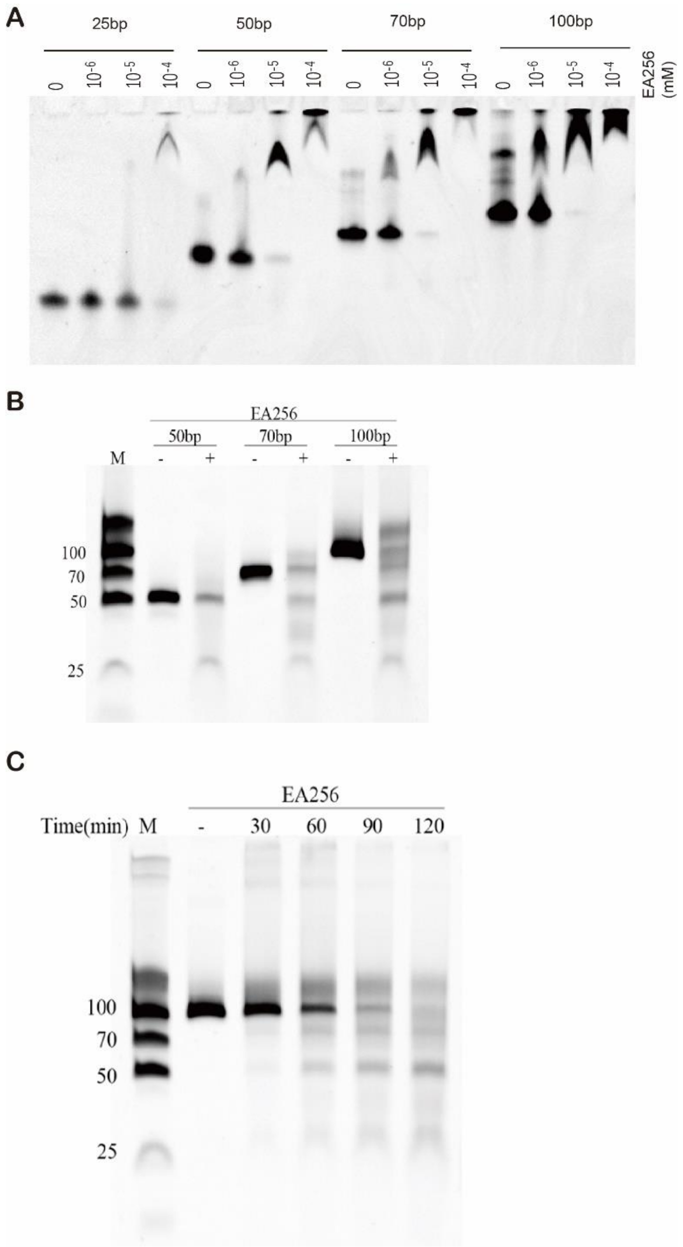

Supplementary Figure S7. (**A**) Original image of cropped gels used in Fig. 6B. (**B**) Original image of cropped gels used in Fig. 6C. (**C**) Original image of cropped gels used in Fig. 6D.

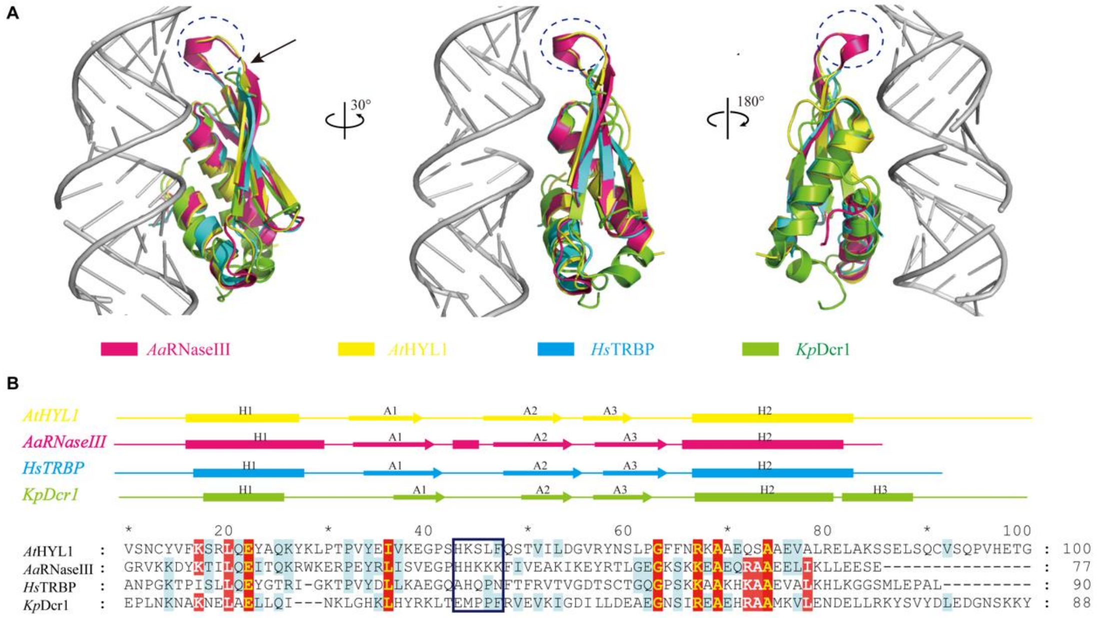

Supplementary Figure S8. (**A**) Structural superposition of dsRBDs. dsRBD from *Aa*RNaseIII, *Arabidopsis thaliana* (*At*) HYL1(PDB Code: 3ADG), *Homo sapiens* (*Hs*) TRBP(PDB Code: 3LLH), and *Kp*Dcr1 is colored in violet, yellow blue, and green, respectively. The loops inserted into the major grooves of dsRNAs are highlighted with dashed ovals. (**B**) Structure based sequence alignment of dsRBDs. The secondary structures are shown on the top. The loop regions interacting with dsRNAs are highlighted with blue box.

Supplementary Table S1. Sequence of the DNA primes and RNAs used in this work.

Name	Sequence (5´→3´)
*Eh*RNaseIII-BamHI-1F	GACTGGATCCAGCTCAACTACATTACACAATGCAATGC
*Eh*RNaseIII-SalI-256R	GACTGTCGACTTATTGTGATGGATGAACAAAAGAATTCC
*Eh*RNaseIII-SalI-229R	GCATGTCGACTTAATCAGAACTACTTAATATATAATCAATATCATTAGTATCAACTTC
*Eh*RNaseIII-SalI-194R	GCATGTCGACTAAATCAGTTTGATATTTTTCTAATGAACTTCTATAATAA
*Eh*RNaseⅢ-E119Q-F	AAAGGAGATTTAGTTCAAAGTTTAATTGGTGGT
*Eh*RNaseⅢ-E119Q-R	TTTCCTCTAAATCAAGTTTCAAATTAACCACCA
*Eh*RNaseIII-256-R-Aa	TTACTCTTCCCTCTTGTGATGGATGAACAAAAGAATTCCA
*Aa*dsRBD-145F	CATCCATCACAA GAGGGAAGAGTAAAAAAAGATTACAAAACG
*Aa*dsRBD-221-SalI-R	GACTGTCGAC TCATTCTGATTCCTCCAGTAATTTAATTAATTC
25 sense	GGGAGAAACGCGGCCTCTTTCTCCC
25 antisense	GGGAGAAAGAGGCCGCGTTTCTCCC
50 sense	GGGAGAAACGCGGCCTCTTCTTATTTATGGCGACATGTTGTGCCTCTCCC
50 antisense	GGGAGAGGCACAACATGTCGCCATAAATAAGAAGAGGCCGCGTTTCTCCC
70 sense	GGGAGAAACGCGGCCTCTTCTTATTTATGGCGACATGTTGTGCCACATATTGAGC CAGTAGCGCTCTCCC
70 antisense	GGGAGAGCGCTACTGGCTCAATATGTGGCACAACATGTCGCCATAAATAAGAAG AGGCCGCGTTTCTCCC
100 sense	GGGAGAAACGCGGCCTCTTCTTATTTATGGCGACATGTTGTGCCACATATTGAGC CAGTAGCGCGGTGTATTATACCAGACCTTATTGGTATGGTCTCCC
100 antisense	GGGAGACCATACCAATAAGGTCTGGTATAATACACCGCGCTACTGGCTCAATATG TGGCACAACATGTCGCCATAAATAAGAAGAGGCCGCGTTTCTCCC
